# Transcriptome-wide association study of HIV-1 acquisition identifies *HERC1* as a susceptibility gene

**DOI:** 10.1016/j.isci.2022.104854

**Published:** 2022-08-04

**Authors:** Rodrigo R.R. Duarte, Oliver Pain, Robert L. Furler, Douglas F. Nixon, Timothy R. Powell

**Affiliations:** 1 Department of Social, Genetic & Developmental Psychiatry, Institute of Psychiatry, Psychology & Neuroscience, King’s College London, 16 De Crespigny Park, London, SE5 8AF, UK; 2 Division of Infectious Diseases, Department of Medicine, Weill Cornell Medicine, Cornell University, New York, NY, 10021, USA

**Keywords:** Genetics, Immunity, Bioinformatics

## Abstract

The host genetic factors conferring protection against HIV type 1 (HIV-1) acquisition remain elusive, and in particular the contributions of common genetic variants. Here, we performed the largest genome-wide association meta-analysis of HIV-1 acquisition, which included 7,303 HIV-1-positive individuals and 587,343 population controls. We identified 25 independent genetic loci with suggestive association, of which one was genome-wide significant within the major histocompatibility complex (MHC) locus. After exclusion of the MHC signal, linkage disequilibrium score regression analyses revealed a SNP heritability of 21% and genetic correlations with behavioral factors. A transcriptome-wide association study identified 15 susceptibility genes, including *HERC1*, *UEVLD*, and *HIST1H4K*. Convergent evidence from conditional analyses and fine-mapping identified *HERC1* downregulation in immune cells as a robust mechanism associated with HIV-1 acquisition. Functional studies on *HERC1* and other identified candidates, as well as larger genetic studies, have the potential to further our understanding of the host mechanisms associated with protection against HIV-1.

## Highlights

• HIV-1 acquisition is highly polygenic, with a SNP heritability estimate of 21%
• HIV-1 acquisition correlates with behavioral factors such as smoking
• *HERC1* downregulation in immune cells is associated with HIV-1 acquisition risk

## Graphical abstract

**Figure:**
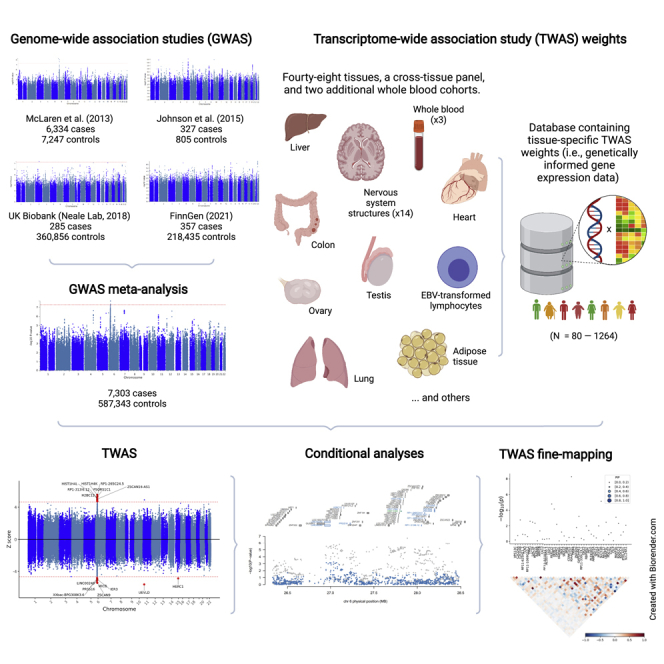

## Introduction

HIV type 1 (HIV-1) acquisition is a complex trait that depends on environmental and genetic factors, including dose of viral inoculum (Rodger et al., 2019) and host behavioral, cellular, and immune parameters moderating susceptibility to infection, viral control, and a systemic spread (Powell et al., 2020). Although there have been many advances in the development of successful prevention options (i.e., pre- and post-exposure prophylaxis [PrEP/PEP] [Donnell et al., 2014; Grant et al., 2010; Sultan et al., 2014]), there is no effective vaccine to prevent a systemic infection. Ultimately, advances in prevention science require a far greater understanding of the molecular mechanisms underlying susceptibility to HIV-1.

There are several host factors associated with HIV-1 acquisition, including cytokines, *CCR5*, *APOBEC3G*, *TRIM5*, human leukocyte antigen (HLA) class I genes, and others (Lama and Planelles, 2007; Martin and Carrington, 2013). However, most studies so far have been unable to distinguish causation from consequence of infection or correlation with co-infections. The best established genetic marker of protection against infection is the *CCR5* *Δ32* homozygous mutation (Alkhatib, 2009), which results in a faulty coreceptor that stops HIV-1 from entering its target cells (Lopalco, 2010). Nevertheless, homozygotes are rare, representing 1.2% of Europeans and less than 0.1% of individuals from other populations (Auton et al., 2015). Because studies prior to antiretroviral therapy have showed that up to two-thirds of individuals exposed to HIV-1 may not become infected despite exposure (Fowke et al., 1996; The Working Group on Mother-To-Child Transmission of HIV, 1995), it is plausible that there are common genetic variants associated with protection against infection. Yet, the molecular mechanisms underlying host susceptibility to infection, particularly those associated with common genetic variants, remain largely unknown.

Genome-wide association studies (GWAS) have the potential to fast-track the identification of common genetic variants and, consequently, genes and biological processes involved in HIV-1 acquisition. Although there was a lack of large genetic studies monitoring at-risk populations prior to the development of antiretroviral therapy, cross-sectional studies analyzing genetic differences between HIV-1-positive and HIV-1-negative individuals are likely to highlight relevant genetic mechanisms of acquisition. However, because associated variants are often noncoding and located in regions of complex linkage disequilibrium, it is challenging to pinpoint the genes directly affected by the risk variants identified in GWAS. In addition, the genetic architecture underlying complex traits originates from multiple tissues, and therefore, GWAS results alone cannot directly inform how or where the risk signal imparts its effects on gene expression regulation. In this context, transcriptome-wide association studies (TWAS) are effective tools to establish how genetic susceptibility exerts its effect (i.e., up- or downregulation of susceptibility genes) in a tissue-informed manner (Gusev et al., 2016, 2018; Mancuso et al., 2017, 2018). Because genetic variants could impinge upon the regulation of key host molecules moderating viral entry or replication (e.g., proteins that act as viral receptors or DNA repair enzymes that degrade foreign nucleic acid material), identifying such regulatory mechanisms associated with HIV-1 acquisition could provide insight into future research on drug and vaccine development. Furthermore, considering that HIV-1 acquisition genetics likely encompasses immune and behavioral factors (Powell et al., 2020), TWAS can help isolate immune processes by identifying *cis*-regulatory mechanisms that more strongly explain the genetic association signal in lymphocytes or whole blood relative to other tissues (e.g., brain tissue). Finally, TWAS aggregate SNP associations for each gene in a functionally informed manner. Because they comprise of gene-level analyses, TWAS reduce the multiple testing burden typically associated with GWAS, and thus, they can be useful in identifying susceptibility genes in less well-powered genetic association studies (Dall’Aglio et al., 2021; Gusev et al., 2016).

The largest GWAS of HIV-1 acquisition to date has identified no common genome-wide significant variants outside of the major histocompatibility complex (MHC) locus (McLaren et al., 2013). However, considering the high estimates of SNP heritability (h^2^_SNP_) observed for HIV-1 acquisition in that study after removing the MHC signal (h^2^_SNP_ = 28–42%) (Powell et al., 2020) and that larger sample sizes typically provide increased power to detect genetic associations (Ziyatdinov et al., 2021), we meta-analyzed their results with findings from Johnson et al. (2015), FinnGen (2021), and the UK Biobank (Neale Lab, 2018). We also performed the first multi-tissue transcriptome-wide association study of HIV-1 acquisition and identified a robust, and arguably the first, common genetic risk mechanism associated with this trait.

## Results

### Genome-wide association meta-analysis of HIV-1 acquisition

Our genome-wide association meta-analysis of HIV-1 acquisition included 7,303 cases and 587,343 controls (total N = 594,646, effective N = 17,014.55). We did not expect any sample overlap across the studies included in the meta-analysis, but we confirmed this through a genetic correlation analysis performed using linkage disequilibrium score (LDSC) regression (Bulik-Sullivan et al., 2015b) (adjusted p > 0.05 for all pairwise comparisons, indicating absence of sample overlap; Table S1).

**Figure 1:**
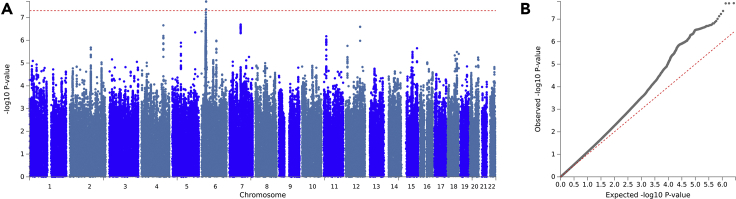
GWAS meta-analysis of HIV-1 acquisition suggests a high degree of polygenicity (A) SNP-level Manhattan plot and (B) quantile-quantile plot of the associations observed. The top associated SNP identified was rs41557415 (p = 1.96 × 10^−8^), located at the MHC locus, on chromosome 6.

Our study assessed the effects of 5,347,926 genetic variants in relation to HIV-1 acquisition. We identified 25 independent loci across the genome with suggestive association (p < 5 × 10^−6^), of which one, at the MHC, surpassed genome-wide significance (p < 5 × 10^−8^) (Figure 1A). Demonstrating the consistency of the signal across the individual studies included in the meta-analysis, we observed that of the 313 variants with suggestive association that were assessed in all four studies, 301 (96%) showed concordant directions of effect across all studies. The distribution of peaks in the Manhattan plot and the deviation of the observed p values from the null hypothesis in the quantile-quantile (Q-Q) plot suggest a high degree of polygenicity associated with HIV-1 acquisition susceptibility (Figure 1B; see more about genomic inflation analyses below, in the sensitivity analyses section). LDSC regression estimated the SNP heritability (h^2^_SNP_) as 0.2105 (0.0369), corroborating a polygenic signal for HIV-1 acquisition.

The top associated SNP identified was rs41557415 (p = 1.96 × 10^−8^), located at the MHC locus, more specifically in the *HLA-B* gene. As expected, this SNP is in linkage disequilibrium with the top association signal from McLaren et al. (2013), rs4418214 (R^2^ = 0.43, D’ = 0.98 in Europeans), which the authors suggest tags HLA-B∗57:01 and 27:05. However, because our meta-analysis was performed using summary statistics only, we were unable to map the MHC signal to a specific HLA haplotype. Further, and as expected, variants working under a recessive genetic model like the *CCR5 Δ32* mutation (or a proxy for it, rs113010081 [R^2^ = 0.94, D’ = 0.98 in Europeans]) did not show an association with HIV-1 acquisition (p > 0.05).

### Genetic correlations with 1,478 complex traits

We investigated traits genetically correlated with HIV-1 acquisition by performing genetic correlation analyses with 1,478 GWAS traits using LD score regression (Bulik-Sullivan et al., 2015a, 2015b; Cuéllar-Partida et al., 2019). We observed associations with traits such as “having ever smoked” (rg = 0.34, p = 4.82 × 10^−8^), “alcohol usually taken with meals” (rg = −0.36, p = 1.69 × 10^−6^), “ever had same-sex intercourse” (rg = 0.62, p = 2.14 × 10^−8^), and “substances taken for depression” (rg = 0.37, p = 2.32 × 10^−5^; all Bonferroni p < 0.05; Table S2).

We calculated the genetic causality proportion (GCP) (O’Connor and Price, 2018) of HIV-1 acquisition on each of the correlated traits separately in an attempt to clarify these relationships (Table S2). Although these analyses produced GCP estimates indicating a low probability of shared causality (|GCP| < 0.60), the top trait pair identified with nominal confidence suggested that “alcohol usually taken with meals” (a sociodemographic indicator) is a protective factor for HIV-1 acquisition (GCP = −0.597, p = 0.036). However, the low-confidence GCP estimates observed for this and other correlated traits after multiple testing correction (Bonferroni p > 0.05) indicate a lack of sufficient evidence to support or dismiss shared genetic causality.

### Functional characterization of the GWAS results

To understand whether there were functional genomic categories enriched within the GWAS results, we performed partitioned heritability using LDSC regression (Table S3). We found that the GWAS results were negatively enriched within a category named “MAF_Adj_LLD_AFR” (coefficient = −185.15 [36.6], p = 4.09 × 10^−4^, Bonferroni p = 0.04). This genomic annotation corresponds to variants whose levels of linkage disequilibrium (LLD) are similar in a reference African population, after adjusting for minor allele frequency (MAF) differences. Thus, a negative enrichment suggests that the associated loci in Europeans will likely have a different LD structure in Africans, thus warranting studies in more diverse populations.

We also tested the GWAS results for enrichment with gene sets involved in known biological pathways and cell types, considering 14,462 biological terms and 209 cell and tissue types. The top gene sets associated with HIV-1 acquisition were “melanoma-associated antigen 1 (MAGEA1) subnetwork” (p = 4.52 × 10^−4^) and “T Lymphocytes Regulatory” (p = 0.024), respectively, but these did not survive multiple testing correction (Bonferroni p > 0.05).

### No significant genetic overlap between HIV-1 acquisition and HIV-1 viral control

To investigate whether the genetic mechanisms underlying susceptibility to HIV-1 acquisition were shared with those regulating viral control in HIV-1-positive individuals, we tested whether PRS for HIV-1 acquisition was associated with viral load in blood, using a cohort of 288 Europeans. We found a nominal genetic overlap between HIV-1 acquisition and viral control, with the most significant effect at the p-value threshold (P_T_) of 0.1 (β = −1297.07, S.E. = 601.37, p = 0.03), which explained 1.6% of the variance in viral load in that cohort. However, this effect did not survive multiple testing correction for the number of thresholds tested (n = 8; adjusted p > 0.05), suggesting a nonsignificant and minimal overlap between polygenic risk for HIV-1 acquisition and viral control in people living with HIV-1.

### Transcriptome-wide association study of HIV-1 acquisition

**Figure 2:**
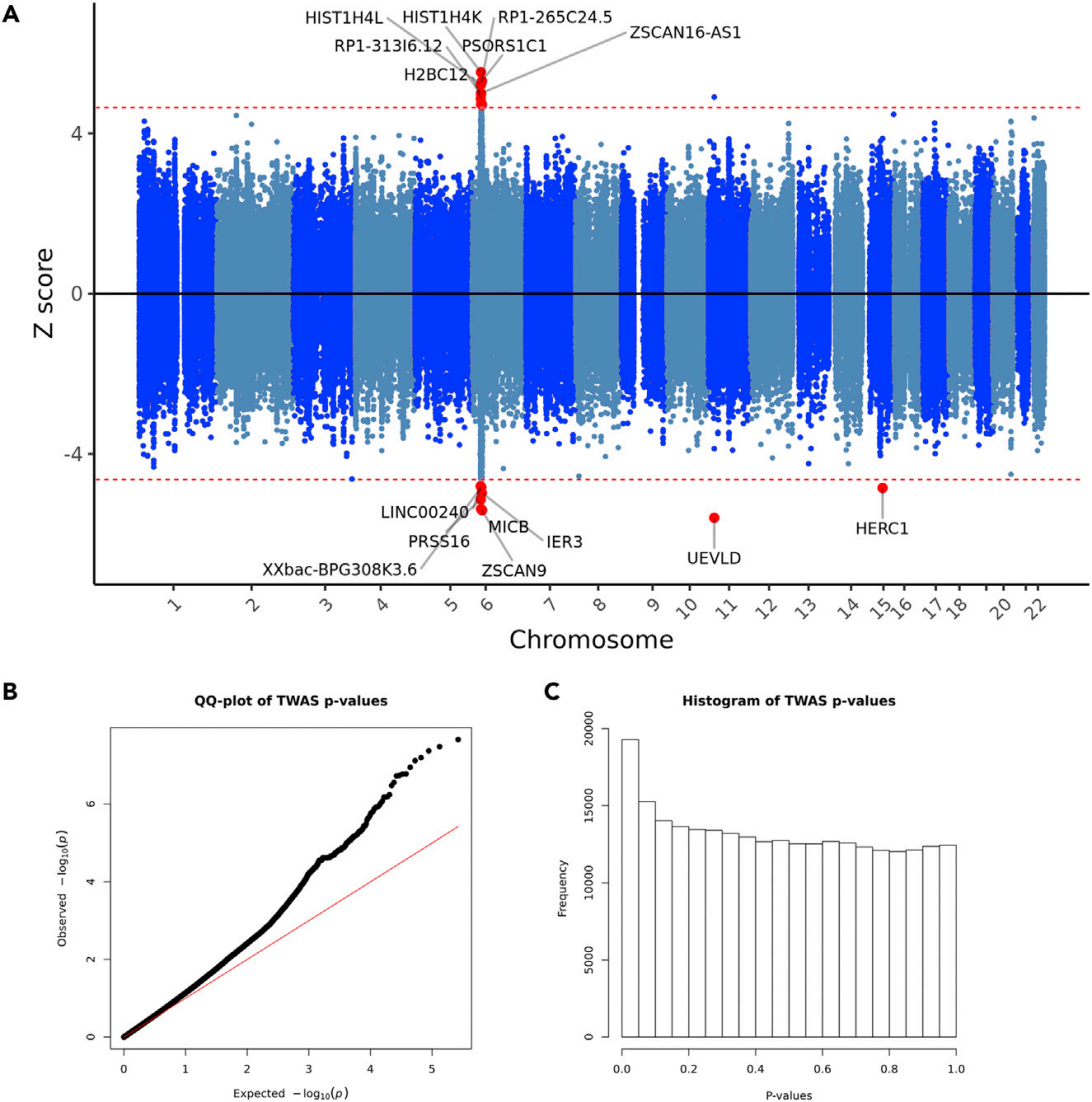
Multi-tissue transcriptome-wide association study of HIV-1 acquisition (A) Manhattan biplot showing all TWAS associations identified, (B) quantile-quantile plot of the associations identified, and (C) distribution of p-values. We observed 15 genes whose expression correlated with HIV-1 acquisition susceptibility, including genes located outside of the MHC complex, such as *UEVLD* (p = 2.18 × 10^−8^, Bonferroni p = 6.00 × 10^−4^), and *HERC1* (p = 1.23 × 10^−6^, Bonferroni p = 0.04).

**Figure 3:**
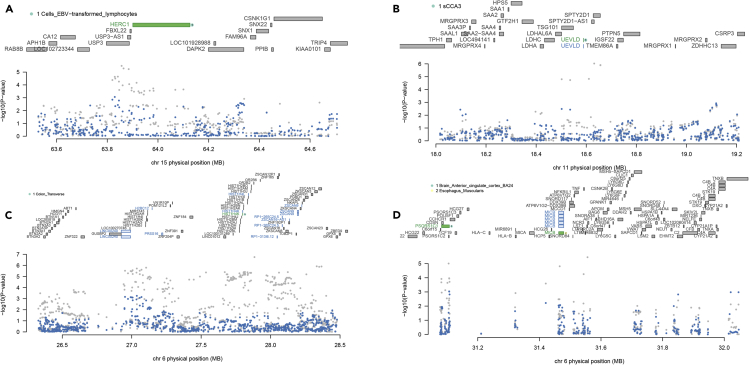
Regional association plots of the TWAS hits Conditional analyses corroborate independent TWAS associations for (A) *HERC1* in EBV-transformed lymphocytes (rs6494412 association before conditioning, p = 3.30 x 10^-6^; after, p = 0.06), (B) *UEVLD* in one of the cross-tissue panels (rs7942526 association before conditioning, p = 9.40 x 10^‑7^; after, p = 0.59), and (C) *HIST1H4K* in the colon transverse (rs13218875 association before conditioning, p = 1.80 x 10^-7^; after, p = 0.0504). On the other hand, (D) *MICB* expression in the esophagus muscularis and *PSORS1C1* in the anterior cingulate cortex explain the GWAS association signal at their locus only in part (rs1265099 association before conditioning, p = 9.70 x 10^-6^; after, p = 0.03). The dots colored in gray and blue correspond to the degree of association of individual SNPs with HIV-1 acquisition, before and after conditioning their association on the predicted expression of the gene(s) highlighted in green at each locus, respectively. The genes highlighted in blue correspond to marginally associated genes.

To identify gene expression profiles associated with susceptibility to HIV-1 acquisition, we performed a multi-tissue transcriptome-wide association study using FUSION (Gusev et al., 2016) (Figure 2A; see TWAS expression weights tested in Table S4). The p-value distribution and quantile-quantile plot suggest that there are many genes whose expression are regulated in association with susceptibility to HIV-1 acquisition (Figures 2B and 2C). After correcting for the number of unique features tested across all TWAS weight panels (27,540 unique genes), we observed 26 Bonferroni-significant TWAS association signals spanning 15 genes and 22 expression panels (p < 0.05/27,540 or 1.82 × 10^−6^; Table S5). The most significant feature was the gene *UEVLD*, from chromosome 11p15.1, in one of the cross-tissue modules (Z = −5.60, p = 2.18 × 10^−8^, Bonferroni p = 6.00 × 10^−4^). Another significant gene was *HERC1*, on chromosome 15q22.31, whose downregulation in EBV-transformed B lymphocytes was associated with HIV-1 acquisition (Z = −4.85, p = 1.23 × 10^−6^, Bonferroni p = 0.04). Notably, the other 24 association signals corresponded to genes located within the MHC locus. These signals must be interpreted with caution due to the complex LD structure in this region, which can result in associations driven by correlation with other *cis*-regulatory mechanisms. We performed conditional analyses in FUSION, where GWAS associations were re-evaluated after controlling for the expression of TWAS-significant genes within their respective loci. Out of the 26 TWAS association signals, only three represented independent associations with HIV-1 acquisition (*HERC1*, *UEVLD*, *HIST1H4K*), whereas the remainder correlated with the expression of these three genes or were unable to explain the GWAS signal in their respective locations. For instance, we observed that the top GWAS signal on chromosome 15, rs6494412 (GWAS p = 3.30 × 10^−6^), was no longer significantly associated with HIV-1 acquisition after controlling for the expression of *HERC1* in EBV-transformed B lymphocytes (conditional p = 0.06; Figure 3A). Similarly, the top GWAS SNP on chromosome 11, rs7942526 (GWAS p = 9.40 × 10^−7^) was not associated with HIV-1 acquisition after controlling for the expression of *UEVLD* in the cross-tissue weights (conditional p = 0.59; Figure 3B). For the MHC region, we observed that the top GWAS hit at the *HIST1H4K* locus, rs13218875 (GWAS p = 1.80 × 10^−7^), was also no longer significantly associated with HIV-1 acquisition after controlling for the expression of that gene (conditional p = 0.0504; Figure 3C). On the other hand, the top GWAS hit at a neighboring MHC location, rs1265099 (GWAS p = 9.70 × 10^−6^), remained significant after controlling for the expression of *MICB* in the esophagus muscularis and *PSORS1C1* in the anterior cingulate cortex (conditional p = 0.03; Figure 3D), which suggests that other genes or regulatory mechanisms (e.g., *trans*-regulatory effects) at this locus may be involved in susceptibility. Ultimately, these findings corroborate a role for *HERC1*, *UEVLD*, and *HIST1H4K* in HIV-1 acquisition.

### TWAS fine-mapping

We used FOCUS to calculate 90%-credible gene sets (i.e., sets likely to contain causal genes) and probability estimates of causality (PIP) for each gene. We observed that *HERC1* expression in EBV-transformed B lymphocytes (PIP = 0.917) and *RTP4* in the aorta (PIP = 0.688) were associated with high estimates of causality (i.e., PIP >0.50). The only gene expression mechanism robustly associated with HIV-1 acquisition according to the conditional analyses from FUSION and the FOCUS fine-mapping analysis was the downregulation of *HERC1*, as identified in EBV-transformed B lymphocytes.

### *HERC1* expression

**Figure 4:**
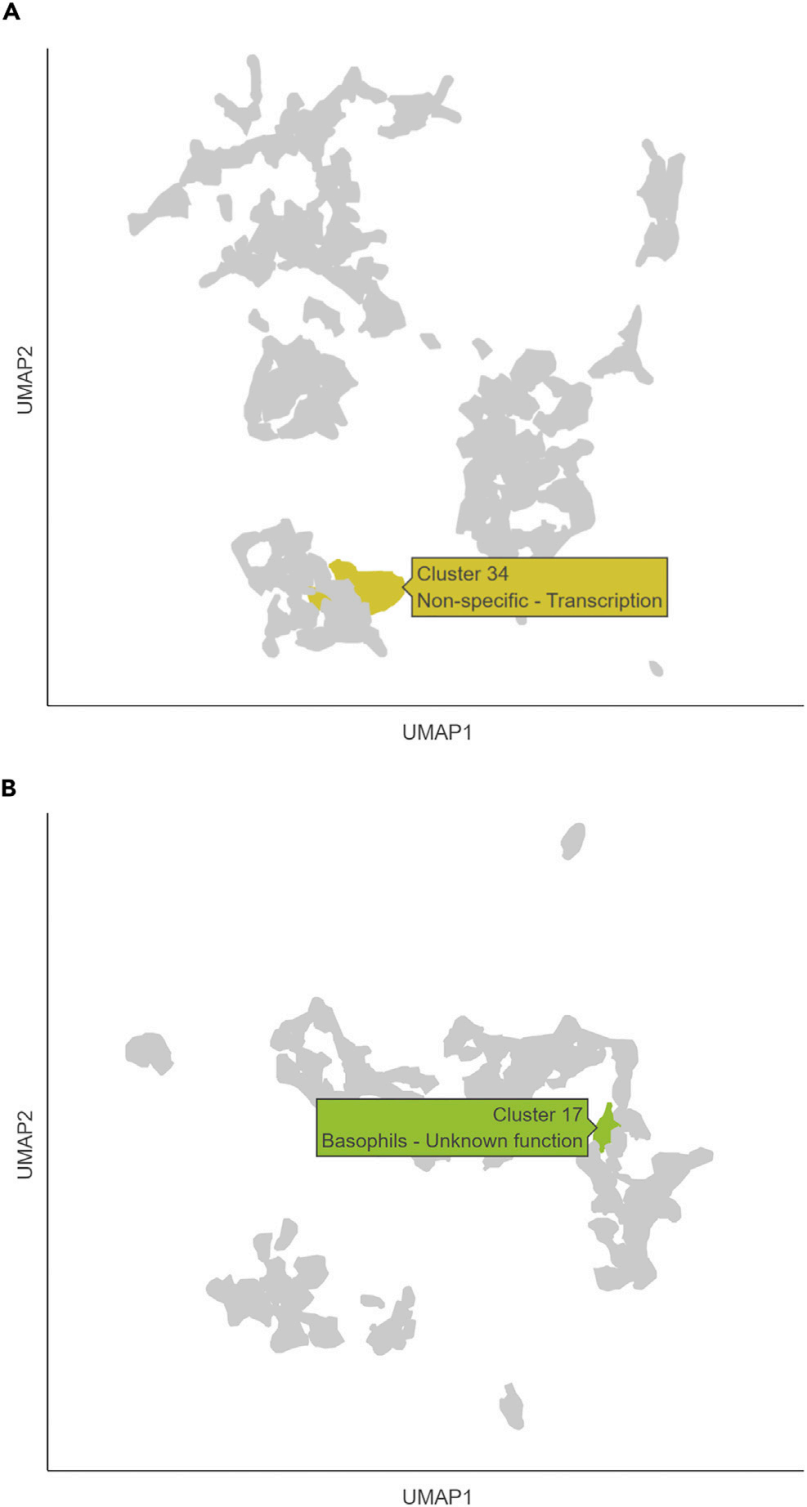
*HERC1* expression according to the Human Protein Atlas Despite the ubiquitous expression of HERC1 in all tissues and cell types, (A) a multi-tissue clustering analysis indicates that *HERC1* expression is likely related to the regulation of transcription. (B) Analysis of immune cell types only showed that *HERC1* expression clustered with genes associated with basophils. UMAP, uniform manifold approximation and projection, used in clustering analysis for dimension reduction.

Analysis of *HERC1* expression in the Human Protein Atlas (Uhlén et al., 2015) showed that *HERC1* was detected in virtually all cells and tissues examined. A clustering analysis suggests, with maximum statistical confidence, that *HERC1* expression is associated with genes involved in transcription regulation (Figure 4A). Among blood cell types, *HERC1* clustered, also with maximum confidence, with genes associated with basophils (Figure 4B).

### Sensitivity analyses

To ensure that there were no systematic biases in our GWAS meta-analysis, we analyzed it using LDSC regression (Bulik-Sullivan et al., 2015b). This analysis produced a genomic inflation factor (*λ*_GC_) estimate of 1.14 with an intercept of 1.07 (0.0077), indicating that there could be an inflation signal due to population stratification in one of the studies included. In fact, LDSC regression of the McLaren study, the largest study included in the meta-analysis, corresponding to approximately 80% of the final effective sample size, produced a *λ*_GC_ estimate of 1.1491 with an intercept of 1.0833 (0.0076). Although the authors did not find evidence of population stratification in their study, we corrected their summary statistics using the intercept genomic control (intercept-GC) method (Bulik-Sullivan et al., 2015b). We meta-analyzed the corrected summary statistics with the other three studies (i.e., Johnson et al. (2015), FinnGen (2021), UK Biobank [Neale Lab, 2018]). This new meta-analysis, when analyzed by LDSC regression, produced a *λ*_GC_ estimate of 1.0649 with an intercept of 1.0031 (0.0072), suggesting that applying the intercept-GC method to the McLaren study alone was sufficient to remove any potential bias in the meta-analysis (i.e., intercept closer to 1). Even though heritability estimates are downward biased after genomic control, we observed that the h^2^_SNP_ of this new meta-analysis remained high (h^2^_SNP_ = 0.197 [0.0337]), corroborating the high degree of polygenicity associated with HIV-1 acquisition. A new TWAS was performed, where we replicated the TWAS associations observed previously: *UEVLD* in the cross-tissue module (Z = −5.40, p = 6.53 × 10^−8^), *HERC1* in EBV-transformed B lymphocytes (Z = −4.70, p = 2.54 × 10^−6^), and *HIST1H4K* in the colon transverse (Z = 5.35, p = 8.77 × 10^−8^) (Table S5). In the FOCUS analysis, the agnostic TWAS fine-mapping identified *HERC1* expression in EBV-transformed B lymphocytes (PIP = 0.858) and *RTP4* in the aorta (PIP = 0.568) as robustly associated with HIV-1 acquisition.

## Discussion

Exposure to infectious agents does not always lead to a systemic infection. For instance, epidemiological studies prior to antiretroviral therapy indicated that up to two-thirds of individuals exposed to HIV-1 do not become infected (Fowke et al., 1996; The Working Group on Mother-To-Child Transmission of HIV, 1995). Although dose of viral inoculum (Pedraza et al., 1999) and route (Patel et al., 2014) of exposure are strong predictors of a systemic infection, it has been hypothesized that host genetic differences also moderate susceptibility to viral entry, replication, and a systemic spread (Limou et al., 2009; Liu et al., 1996; McLaren et al., 2013, 2015; Powell et al., 2020). However, aside from *CCR5*, the host genetic factors involved in susceptibility to HIV-1 acquisition, particularly those related to common genetic variants, remain elusive. Here, we performed the largest genome-wide association meta-analysis and the first multi-tissue TWAS of HIV-1 acquisition to advance our understanding of the genetic factors involved in this trait.

The GWAS identified 25 independent loci with suggestive association, of which one, at the MHC, surpassed genome-wide significance. Although the MHC remains a fundamental target for HIV-1 research (e.g., Pereyra et al., 2010), we were unable to confidently infer protective MHC haplotypes, given the current lack of methods to do this using GWAS summary statistics alone. This is a challenging task considering the large linkage disequilibrium block sizes located in this highly polymorphic region (Dawkins and Lloyd, 2019). However, in our study, we explored the polygenic architecture of HIV-1 acquisition using genetic correlations, partitioned heritability, gene-set enrichment analyses, and a TWAS. This genome-wide characterization of HIV-1 acquisition was needed considering the high h^2^_SNP_ estimated for this trait using the LD score regression method, which disregards SNPs located at the MHC to avoid inflated estimates (Bulik-Sullivan et al., 2015b). Thus, our study represents an important step toward a better understanding of the risk genes involved in HIV-1 acquisition, beyond those explored due to historical relevance alone, which could reveal genuine mechanisms associated with susceptibility, identified through hypothesis-free analyses, as observed in other fields (e.g., psychiatry, see Duarte et al., 2021).

We found that HIV-1 acquisition was associated with a h^2^_SNP_ estimate of 0.21 (0.04), which represented a 25% reduction in heritability relative to what we observed previously in the McLaren study alone (h^2^_SNP_ = 0.28 [0.05]) (Powell et al., 2020). Although increases in sample size improve the power to detect the core genes associated with a GWAS trait, it is plausible that the individual studies included in the meta-analysis also increased the complexity of the phenotype analyzed because of the different case-control selection criteria used in each study (increased phenotype complexity typically decreases heritability estimates; see Tropf et al., 2017). For instance, it is plausible that the Johnson et al. (2015) study, in which cases and controls were matched injecting drug users, may provide insight into the genetic signals that more strongly reflect immune processes. On the other hand, studies analyzing cases versus population controls, such as the McLaren et al. (2013) study, may highlight genetic signals that reflect more socioeconomic or behavioral factors. The observed reduction in heritability is also consistent with the lower estimates of h^2^_SNP_ calculated for susceptibility to infections in general (∼2%–7% [Nudel et al., 2019]).

We observed genetic correlations between HIV-1 acquisition and multiple traits that were previously identified in a study from our group, where we performed LD score regression analyses on the McLaren study alone (Powell et al., 2020). For example, we confirmed an association with smoking status, which is consistent with epidemiological findings showing that smoking is more prevalent among HIV-1-positive individuals relative to the general population (Batista et al., 2013). Another correlated trait was “alcohol usually taken with meals,” which is a proxy for socioeconomic status (Zhou et al., 2021). It is plausible that this is a reflection of the fact that infection is typically higher among individuals with lower income and fewer years of education (Bunyasi and Coetzee, 2017). As a reflection of the more extensive GWAS catalog used in the present screening, we further identified new traits associated with HIV-1 acquisition, including “ever had same-sex intercourse” and “substances taken for depression.” These results corroborate known risk factors associated with HIV-1 acquisition, i.e., there is an increased prevalence of HIV-1 among men who have sex with men (Center for Disease Control and Prevention, 2021) and an increased prevalence of depression in people living with HIV-1 (Tran et al., 2019).

To identify the gene expression profiles associated with HIV-1 acquisition, we performed a multi-tissue TWAS. The TWAS identified 15 genes regulated in association with HIV-1 acquisition, of which three genes had their expression considered independent from other genes in their loci (*UEVLD*, *HERC1*, and *HIST1H4K*). None of these genes were identified in a recent TWAS of HIV-1 viral control (Li et al., 2021). Using an agnostic TWAS fine-mapping approach, we found evidence corroborating an association between HIV-1 acquisition and *HERC1* expression in EBV-transformed B lymphocytes and *RTP4* expression in the aorta. Our findings pertaining to *HERC1*, *UEVLD*, *HIST1H4K*, and *RTP4*, suggest these genes are important candidates for future research. In fact, the RTP4 protein is a potent inhibitor of pathogenic viruses from the *Flaviviridae* family (Boys et al., 2020), whereas UEVLD has been found in urinary extracellular vesicles from HIV-1-positive individuals (Anyanwu et al., 2018), and HIST1H4K is a histone shown to bind to HIV-1 Tat (Deng et al., 2001). However, *HERC1*, which encodes a large ubiquitin ligase protein, was the most interesting candidate identified in our study.

We obtained converging evidence from FUSION’s conditional analysis and the FOCUS fine-mapping approach suggesting *HERC1* downregulation in EBV-transformed B lymphocytes is robustly associated with HIV-1 acquisition. However, the role of *HERC1* in HIV-1 biology remains unclear, particularly in the context of B lymphocytes. Abnormal levels of B lymphocytes are a characteristic of HIV-1 infection (Liechti et al., 2019), but it is plausible that we detected this *cis*-regulatory effect in EBV-transformed B lymphocytes merely because these cells comprised the only expression dataset in the TWAS consisting of a single immune cell type. Considering that typically 50% of common variants are associated with gene expression regulation in any tissue (GTEx Consortium et al., 2017), it is likely that this regulatory effect also extends to other immune cell types, such as basophils, where *HERC1* appears to be more highly expressed. *HERC1* is known to regulate the formation of infectious synapses transferring viruses from basophils to T cells, which are crucial for the establishment of a systemic infection (Jiang et al., 2015). Downregulation of this gene is also associated with activation of ERK signaling (Schneider et al., 2018), which is known to decrease the production of the antiviral cytokine IFN-γ (Zhang et al., 2018) and to increase HIV-1 infectivity *in vitro* due to differential phosphorylation of HIV-1 components Vif, Rev, and Tat (Yang and Gabuzda, 1999). The exact function of *HERC1* in relation to HIV-1 susceptibility, however, requires further study using functional approaches.

Our study highlights regulatory mechanisms associated with HIV-1 acquisition and one in particular pertaining to the downregulation of *HERC1* in immune cells. Analysis of larger genetic cohorts and functional studies of the genes highlighted here are likely to help uncover host mechanisms moderating HIV-1 acquisition, which could ultimately help us to identify novel approaches to tackle and eradicate HIV-1.

### Limitations of the study

There are limitations to our study that should be acknowledged. First, the effective sample size of our current study is still limited and represents individuals of European ancestry only, and therefore the analysis of larger and more diverse cohorts is likely to provide additional insight; this is particularly relevant, as we found that the associated loci in Europeans likely have a different LD structure in Africans, and the functional implications of this observation remain unclear. Second, we explored HIV-1 acquisition genetics using a cross-sectional analysis of cases and controls defined based on self-report or immunoreactivity, as opposed to studying at-risk individuals longitudinally using immunoassay tests. Although the latter study design could be better at identifying genetic variants regulating specific immunological mechanisms associated with HIV-1 acquisition, it would also represent a more challenging approach (cohorts likely to be smaller, study likely to be more time-consuming), which is also ethically questionable (following at-risk individuals without ensuring they receive PrEP). Although cross-sectional studies may be influenced by misclassification bias (i.e., population controls are still susceptible to infection) (McLaren and Fellay, 2021), we chose this study design and case-control selection because it would allow us to draw power from large population genetic studies to detect more genetic associations. Ultimately, it is plausible that revisiting historical samples from at-risk individuals, collected prior to the development of PrEP, may be a useful way to advance our understanding of HIV-1 acquisition genetics. Third, our TWAS approach assesses only the *cis*-genetic component of gene expression, and future studies should investigate other regulatory mechanisms (e.g., *trans*-eQTL effects) associated with HIV-1 acquisition. Finally, although we shed light on the relationship between susceptibility to HIV-1 acquisition and regulated genes, we cannot infer a causal mechanism yet. Functional studies investigating the expression of genes identified here, particularly *HERC1* levels in lymphocytes and basophils, in relation to viral susceptibility *in vitro,* will be crucial to understanding this relationship.

## STAR★Methods

### Key resources table

**Table:** 

REAGENT or RESOURCE	SOURCE	IDENTIFIER
**Deposited Data**		

GWAS summary statistics from this study	This manuscript; dbGap: phs000454.v1.p1	https://doi.org/10.18742/18166406.v1
GWAS summary statistics from McLaren et al 2013; 6,334 HIV-1-positive cases defined based on serum immunoreactivity and 7,247 population controls	Provided by the authors upon request	https://doi.org/10.1371/journal.ppat.1003515
GWAS summary statistics from Johnson et al 2015; 327 HIV-1-positive cases and 805 HIV-1-negative controls, defined based on serum immunoreactivity	Provided by the authors upon request	https://doi.org/10.1371/journal.pone.0118149 dbGap: phs000454.v1.p1
GWAS summary statistics from UK Biobank; 285 HIV-positive cases and 360,856 population controls, defined based on self-report	Neale Lab, publicly available	Data release 3, trait ID "20002_1439 HIV/AIDS", from https://docs.google.com/spreadsheets/d/1kvPoupSzsSFBNSztMzl04xMoSC3Kcx3CrjVf4yBmESU/edit#gid=227859291
GWAS summary statistics from FinnGen; 357 HIV-positive cases and 218,435 population controls, defined based on self-report	FinnGen, publicly available	Data release 5, trait ID "AB1_HIV", from https://console.cloud.google.com/storage/browser/finngen-public-data-r5/summary_stats

**Software and Algorithms**		

LDSC's munge_sumstats.py, ldsc.py, reference Hapmap3 SNPs, partitioned heritability method	Finucane et al., 2015	https://github.com/bulik/ldsc
liftOver	Hinrichs et al., 2006	https://genome.sph.umich.edu/wiki/LiftOver
METAL	Willer et al., 2010	https://genome.sph.umich.edu/wiki/METAL_Documentation
Plink 1.9	Chang et al., 2015	https://www.cog-genomics.org/plink/
FUMA	Watanabe et al., 2017	https://fuma.ctglab.nl/
FUSION, genotype data corresponding to the European subset of the 1000 Genomes cohort, TWAS weights	Gusev et al., 2016	https://gusevlab.org/projects/fusion/
The Genetics of Complex Traits Browser analysis for gene set enrichment analysis, genetic correlations and genetic causal proportion calculations	Cuéllar-Partida et al., 2019	https://genoma.io/
FOCUS	Mancuso et al., 2019	https://github.com/bogdanlab/focus
PRSice-2	Choi and O'Reilly, 2019	https://www.prsice.info/
Human Protein Atlas	Uhlén et al., 2015	https://proteinatlas.org/
R 3.6.1	The R Project for Statistical Computing, Vienna, Austria	https://www.r-project.org/
All code used in the manuscript	This manuscript	https://doi.org/10.18742/20343219

### Resource availability

#### Lead contact

Further information and requests for resource sharing should be directed to and will be fulfilled by the lead contact, Rodrigo Duarte (rodrigo.duarte@kcl.ac.uk).

#### Materials availability

This study did not generate new unique reagents or material.

### Method details

#### GWAS summary statistics

We define HIV-1 acquisition as a binary phenotype corresponding to whether an individual is currently diagnosed as HIV-1-positive or not. We identify genetic variants associated with HIV-1 acquisition by investigating genetic differences between HIV-1-positive and HIV-1-negative individuals, defined based on immunoreactivity or self-report. To perform the meta-analysis, we downloaded summary statistics from FinnGen (2021) (data release 5, trait ID AB1_HIV, 357 cases and 218,435 population controls, defined based on self-report), and from the UK Biobank (Neale Lab, 2018) (from the Neale lab, data release 3, trait ID 20002_1439 HIV/AIDS, 285 cases and 360,856 population controls, defined based on self-report; see key resources table). These cohorts contribute to less than 10% of the cases in our meta-analysis, and whilst no distinction was made between HIV-1 and HIV-2 in these studies, cases are assumed to be HIV-1-positive since 95% of HIV infections worldwide are attributed to HIV-1 (Campbell-Yesufu and Gandhi, 2011; Gottlieb et al., 2018). We also obtained GWAS summary statistics from studies where cases were confirmed as HIV-1-positive, including Johnson et al. (2015) (327 HIV-1-positive cases and 805 HIV-1-negative controls, defined based on serum immunoreactivity) and McLaren et al. (2013) (6,334 HIV-1-positive cases defined based on serum immunoreactivity and 7,247 population controls), which were kindly provided by the authors of these studies. All cohorts corresponded to individuals of European ancestry. Cases and controls from the Johnson et al. (2015) study were drug users, whereas cases from the McLaren et al. (2013) study, FinnGen (2021) and UK Biobank (Neale Lab, 2018) were likely associated with multiple infection routes.

#### GWAS meta-analysis

Summary statistics from individual studies were pre-processed using custom-made code. We analyzed only non-ambiguous biallelic single nucleotide polymorphisms (SNPs) with minor allele frequency (MAF) > 1%, imputation R^2^ > 0.8 (if information was available), that were located on the autosomes. For the McLaren study (which was a meta-analysis), we isolated only variants that were assessed in all cohorts, since there was no sample size information available per SNP. For all studies, SNPs were renamed using their chromosomal position (hg19), and FinnGen’s summary statistics was lifted to hg19 using liftOver (Hinrichs et al., 2006), for compatibility. The individual studies were analyzed using linkage disequilibrium score (LDSC) regression (Bulik-Sullivan et al., 2015b, 2015c) using default settings, to test for potential sample overlap, analyzing HapMap3 SNPs only. We performed the meta-analysis in METAL (Willer et al., 2010) using a sample size weighted analysis. The effective weight/sample size (N_eff_) per study was calculated using the formula: N_eff_ = 4/(1/N_cases_ + 1/N_controls_) (Willer et al., 2010). We report only SNPs tested in a minimum effective sample size corresponding to half of N_eff_’s 90^th^ percentile (Bulik-Sullivan et al., 2015b), that were also annotated in dbSNP 151. We used PLINK 1.9 (Chang et al., 2015) to identify independent association signals, by clumping variants with p < 1 × 10^−4^ using an R^2^ threshold of 0.25 and a 500 kb window (Lam et al., 2020). Genome-wide significant association was indicated by p < 5 × 10^−8^, whereas suggestive association by p < 5 × 10^−6^ (Coleman et al., 2016b). Manhattan and Q-Q plots were generated using the Functional Mapping and Annotation of Genome-Wide Association Studies (FUMA) tool (Watanabe et al., 2017).

#### GWAS quality control

Estimates of SNP heritability (h^2^_SNP_), genomic inflation (*λ*_GC_), and the intercept were calculated using LDSC regression, following the authors’ manual. As a sensitivity analysis, we applied the intercept genomic control (intercept-GC) method (Bulik-Sullivan et al., 2015b) to the McLaren et al. (2013) study, by multiplying the SNPs’ standard errors by the square root of that study’s intercept. Next, we re-calculated the p values based on the adjusted standard errors and respective betas, and meta-analyzed the corrected summary statistics alongside the other three studies (i.e., Johnson et al., 2015; FinnGen, 2021; UK Biobank [Neale Lab, 2018]), where we repeated our quality control procedure and the downstream processes (e.g., TWAS, conditional analyses, and TWAS fine-mapping).

#### GWAS functional mapping

We performed a partitioned heritability analysis using LDSC regression (Bulik-Sullivan et al., 2015b; Finucane et al., 2015), according to the author’s manual, where only HapMap3 SNPs were analyzed after excluding the MHC locus. LD score weights (baseline model v2.2) and the files corresponding to the European subset of the 1000 Genomes project, used as the reference population, were downloaded from the authors’ website. The baseline model used included 97 genomic annotations corresponding to promoter regions, DNA and histone modification sites, DNase I hypersensitivity sites, conserved regions, and others (Finucane et al., 2015). We also analyzed the GWAS summary statistics using The Genetics of Complex Traits Browser (Cuéllar-Partida et al., 2019), where independent association signals (variants with p < 1 × 10^−5^, clumped using an R^2^ threshold of 0.05 and a 1 Mb window) were tested for enrichment of biological terms using DEPICT and a catalog of 14,462 gene sets (Pers et al., 2015). These sets corresponded to biological annotations reflecting molecular pathways from protein-protein interaction studies, manually curated pathways, and gene sets from mouse knock-out studies. Another enrichment analysis was performed to test the GWAS results for enrichment of genes expressed in specific cell types or tissues, according to 209 Medical Subject Heading (MeSH) annotations derived from 37,427 microarrays (Pers et al., 2015). Only enrichments surviving correction for the number of gene sets tested in each analysis were considered significant (Bonferroni p < 0.05).

#### Transcriptome-wide association study (TWAS)

We ran a TWAS on the autosomes using default settings in FUSION (Gusev et al., 2016), and the TWAS weights calculated by the authors. We used TWAS weights corresponding to 48 tissues available within GTEx (GTEx Consortium et al., 2017) (including whole blood, EBV-transformed B lymphocytes, colon, multiple brain regions, liver, stomach, lungs, ovary, and others; see details in Table S4). We chose this approach since the impact of susceptibility to HIV-1 in terms of gene expression features across tissues and organs had not yet been explored. We also analyzed two additional blood cohorts (the Netherlands Twin Registry (Wright et al., 2014) and the Young Finns Study (Gusev et al., 2016; Raitakari et al., 2008)), which are better powered to detect heritable expression components, relative to the GTEx whole blood sample. Finally, we included three gene expression models corresponding to cross-tissue expression weights combining all heritable gene expression information from across tissues and individuals within GTEx, which improve the detection of heritable *cis* expression mechanisms by TWAS (Feng et al., 2021). We used the 1000 Genomes Phase 3 European panel as LD reference for the TWAS and fine-mapping analysis, downloaded from the FUSION website. Association signals were corrected using the Bonferroni method, considering the number of unique genes tested across all weight panels (P cut-off = 0.05/27,540, or 1.82 × 10^−6^). Plots were generated using the FUSION pipeline and scripts adapted from https://opain.github.io/MDD-TWAS/ (Dall’Aglio et al., 2021). We performed conditional analyses in FUSION to determine whether the significant TWAS associations were independent from the expression of other genes in their respective loci.

#### TWAS fine-mapping

We used FOCUS (Mancuso et al., 2019) to perform TWAS fine-mapping and to narrow down the genes most likely to be causally related to HIV-1 acquisition. FOCUS uses a standard Bayesian approach to define 90%-credible gene sets (gene sets likely to contain causal genes at susceptibility loci) and calculates the posterior inclusion probability (PIP) for each gene in the region to be causal given the observed TWAS statistics. Genes with PIP >0.50 are more likely to be causal for the association than any other gene, or the null model (i.e., when the causal gene is not present in the TWAS). A FOCUS database was created using the TWAS FUSION weights mentioned above (except for the cross-tissue weights, since the objective of the fine-mapping approach was to define relevant gene-tissue pairs). We applied FOCUS across all tissue-specific SNP weight panels simultaneously using an agnostic analysis to identify genes and tissues more likely to be involved in the trait, without potentially biasing the analysis by prioritizing specific tissues.

#### Genetic correlations

We uploaded the GWAS results to The Genetics of Complex Traits Browser (Cuéllar-Partida et al., 2019), where whole-genome correlations using LD score regression (Bulik-Sullivan et al., 2015a, 2015b) were performed against 1478 GWAS traits. Genetic correlation (rg) p values were corrected for multiple testing using the Bonferroni method. Significant trait correlations with HIV-1 acquisition (Bonferroni p < 0.05) were further tested in genetic causal proportion (GCP) analyses to quantify partial causality and directionality of effect (O’Connor and Price, 2018). Resulting GCP values range from 0 (i.e., no genetic causality between HIV-1 acquisition and the correlated trait) to 1 (i.e., full genetic causality), and the sign of this value indicates the direction of the effect (positive GCP: HIV-1 acquisition predisposes to the correlated trait; negative GCP: the correlated trait predisposes to HIV-1 acquisition). GCP p values are considered significant (indicating shared genetic causality) if they survive multiple testing correction for the number of tests made (i.e., the number of traits significantly correlated with HIV-1 acquisition; Bonferroni p < 0.05).

#### Polygenic risk scoring

We performed polygenic risk scoring using PRSice-2 (Choi and O'Reilly, 2019) to test whether there was a shared genetic relationship between HIV-1 acquisition and HIV-1 viral control. We define viral control as a quantitative phenotype corresponding to one’s predisposition to a lower viral load (log_10_ copy/mL) after the establishment of a systemic infection. We generated polygenic risk scores for HIV-1 acquisition at a range of p value thresholds (P_T_ = 0.001, P_T_ = 0.05, P_T_ = 0.1, P_T_ = 0.2, P_T_ = 0.3, P_T_ = 0.4, P_T_ = 0.5, P_T_ = 1) using the summary statistics from our HIV-1 acquisition GWAS meta-analysis, which was performed on individuals of European ancestry. We then tested whether these scores predicted variability in viral load in a subset of the Urban Health Study cohort (dbGap: phs000454.v1.p1) (Johnson et al., 2015), corresponding to individuals of European ancestry who had their viral load measured. European ancestry was confirmed by merging their genotype data with the 1000 Genomes reference panel. Principal components 1 and 2 were generated, and all individuals whose Euclidean distance fell within a defined radius of known Europeans (europeanTh, scaling factor = 1) were confirmed as Europeans using plinkQC (Meyer, 2020), and were further analyzed. We removed individuals with missing genotype data >5%, or variants with missing data >5%, minor allele frequency (MAF) < 0.05, and those with Hardy-Weinberg test p < 1 × 10^−5^ (Coleman et al., 2016a). To control for remaining ancestry differences, we generated ancestry principal components using LD-pruned genotype data in PLINK (Chang et al., 2015). Plotting principal components (PCs) revealed that the first four were sufficient to correct for genetic differences within our sample. We then adjusted viral load values (log_10_ copy/mL) by the first four PCs by taking standardized residuals, generating PC-adjusted Z-scores. After removing outliers (i.e., samples with corresponding Z-scores ±2 standard deviations away from the mean), this sub-cohort consisted of 288 individuals, of which 245 were men, with ages ranging from 20-56 (mean age = 35.34, SD= 7.44). These data were normally distributed (skewness and kurtosis values between −1 and 1) and were not associated with age or sex according to linear regressions (p > 0.05). Z-scores were then input into a phenotype file for polygenic risk scoring analysis in PRSice-2.

#### *HERC1* expression interrogation

To advance our understanding of *HERC1* biology, we investigated its expression profile in the Human Protein Atlas (accessed in June 2022) (Uhlén et al., 2015). This database uses gene expression data from 36 tissues from The Genotype-Tissue Expression (GTEx) consortium and their own biobank (69 cell lines, 52 human tissues and 18 blood cell types), to interrogate gene expression profiles across multiple tissues and cell types. Dimension reduction of the RNA expression data was performed by the authors using the Uniform Manifold Approximation and Projection (UMAP) method. Confidence scores reflecting the confidence of genes assigned to manually curated clusters, which range from zero to one, were calculated by the authors.

#### Statistical analyses

Analyses were performed in Bash (GNU Project Bourne Again SHell) or R 3.6.1 (The R Project for Statistical Computing, Vienna, Austria). Linear regressions were performed in IBM SPSS Statistics version 26 (IBM, Armonk, NY, USA).

## Data Availability

GWAS summary statistics were deposited at Figshare and are publicly available as of the date of publication. DOI is listed in the key resources table. All original code has been deposited at Figshare and is publicly available as of the date of publication. DOI is listed in the key resources table. Any additional information required to reanalyze the data reported in this paper is available from the lead contact upon request.

## Supplemental information

Table S1. Genetic correlation across the different studies analyzed, according to LD score regression, related to Figure 1A

Table S2. Significant genetic correlations (rg) and estimates of causal genetic proportion (GCP) between HIV-1 acquisition and GWAS traits assessed within The Complex Trait Browser, related to Figure 1A

Table S3. Partitioned heritability analysis of HIV-1 acquisition using LD score regression, related to Figure 1A

Table S4. TWAS weights included in the analysis, related to Figure 2A

Table S5. Results summary, including the resulting TWAS, conditional analysis and TWAS fine-mapping, before and after adjusting the McLaren study using the intercept-GC method, related to Figure 2A

## References

[bib1] Alkhatib G. (2009). The biology of CCR5 and CXCR4. Curr. Opin. HIV AIDS.

[bib2] Anyanwu S.I., Doherty A., Powell M.D., Obialo C., Huang M.B., Quarshie A., Mitchell C., Bashir K., Newman G.W. (2018). Detection of HIV-1 and human proteins in urinary extracellular vesicles from HIV+ patients. Adv. Virol..

[bib3] Brooks L.D., Garrison E.P., Durbin R.M., McCarthy S., Abecasis G.R., Chakravarti A., Clark A.G., Donnelly P., Eichler E.E., 1000 Genomes Project Consortium (2015). A global reference for human genetic variation. Nature.

[bib4] Batista J.d.L., Militão de Albuquerque M.d.F.P., Ximenes R.A.d.A., Miranda-Filho D.d.B., Lacerda de Melo H.R., Maruza M., Moura L.V., Pinto da Costa Ferraz E.J.S., Rodrigues L.C. (2013). Prevalence and socioeconomic factors associated with smoking in people living with HIV by sex, in Recife, Brazil. Rev. Bras. Epidemiol..

[bib5] Boys I.N., Xu E., Mar K.B., De La Cruz-Rivera P.C., Eitson J.L., Moon B., Schoggins J.W. (2020). RTP4 is a potent IFN-inducible anti-flavivirus effector engaged in a host-virus arms race in bats and other mammals. Cell Host Microbe.

[bib6] Bulik-Sullivan B., Finucane H.K., Anttila V., Gusev A., Day F.R., Loh P.-R., Perry J.R.B., ReproGen Consortium, Psychiatric Genomics Consortium, Genetic Consortium for Anorexia Nervosa of the Wellcome Trust Case Control Consortium 3 (2015). An atlas of genetic correlations across human diseases and traits. Nat. Genet..

[bib7] Bulik-Sullivan B.K., Loh P.-R., Finucane H.K., Ripke S., Yang J., Daly M.J., Price A.L., Neale B.M., Schizophrenia Working Group of the Psychiatric Genomics Consortium (2015). LD Score regression distinguishes confounding from polygenicity in genome-wide association studies. Nat. Genet..

[bib8] Bulik-Sullivan B.K., Loh P.R., Finucane H.K., Ripke S., Yang J., Daly M.J., Price A.L., Neale B.M., Schizophrenia Working Group of the Psychiatric Genomics Consortium (2015). LD Score regression distinguishes confounding from polygenicity in genome-wide association studies. Nat. Genet..

[bib9] Bunyasi E.W., Coetzee D.J. (2017). Relationship between socioeconomic status and HIV infection: findings from a survey in the free state and Western cape provinces of South Africa. BMJ Open.

[bib10] Campbell-Yesufu O.T., Gandhi R.T. (2011). Update on human immunodeficiency virus (HIV)-2 infection. Clin. Infect. Dis..

[bib11] Center for Disease Control and Prevention (2021). HIV Surveillance Report 32.

[bib12] Chang C.C., Chow C.C., Tellier L.C., Vattikuti S., Purcell S.M., Lee J.J. (2015). Second-generation PLINK: rising to the challenge of larger and richer datasets. Gigascience.

[bib13] Choi S.W., O'Reilly P.F. (2019). PRSice-2: polygenic Risk Score software for biobank-scale data. Gigascience.

[bib14] Coleman J.R.I., Euesden J., Patel H., Folarin A.A., Newhouse S., Breen G. (2016). Quality control, imputation and analysis of genome-wide genotyping data from the Illumina HumanCoreExome microarray. Brief. Funct. Genomics.

[bib15] Coleman J.R.I., Lester K.J., Keers R., Roberts S., Curtis C., Arendt K., Bögels S., Cooper P., Creswell C., Dalgleish T. (2016). Genome-wide association study of response to cognitive–behavioural therapy in children with anxiety disorders. Br. J. Psychiatry.

[bib16] Cuéllar-Partida G., Lundberg M., Kho P.F., D’Urso S., Gutiérrez-Mondragón L.F., Hwang L.D., Hwang L.-D. (2019). Complex-Traits Genetics Virtual Lab: a community-driven web platform for post-GWAS analyses. Preprint at bioRxiv.

[bib17] Dall’Aglio L., Lewis C.M., Pain O. (2021). Delineating the genetic component of gene expression in major depression. Biol. Psychiatry.

[bib18] Dawkins R.L., Lloyd S.S. (2019). MHC genomics and disease: looking back to Go forward. Cells.

[bib19] Deng L., Wang D., de la Fuente C., Wang L., Li H., Lee C.G., Donnelly R., Wade J.D., Lambert P., Kashanchi F. (2001). Enhancement of the p300 HAT activity by HIV-1 Tat on chromatin DNA. Virology.

[bib20] Donnell D., Baeten J.M., Bumpus N.N., Brantley J., Bangsberg D.R., Haberer J.E., Mujugira A., Mugo N., Ndase P., Hendrix C., Celum C. (2014). HIV protective efficacy and correlates of tenofovir blood concentrations in a clinical trial of PrEP for HIV prevention. J. Acquir. Immune Defic. Syndr..

[bib21] Duarte R.R.R., Brentani H., Powell T.R. (2021). Ditching candidate gene association studies: lessons from psychiatric genetics. Braz. J. Psychiatry..

[bib22] Feng H., Mancuso N., Gusev A., Majumdar A., Major M., Pasaniuc B., Kraft P. (2021). Leveraging expression from multiple tissues using sparse canonical correlation analysis and aggregate tests improves the power of transcriptome-wide association studies. PLoS Genet..

[bib23] FinnGen (2021). FinnGen Public Data R5. https://www.finngen.fi/en/access_results.

[bib24] Finucane H.K., Bulik-Sullivan B., Gusev A., Trynka G., Reshef Y., Loh P.-R., Anttila V., Xu H., Zang C., Farh K. (2015). Partitioning heritability by functional annotation using genome-wide association summary statistics. Nat. Genet..

[bib25] Fowke K.R., Nagelkerke N.J., Kimani J., Simonsen J.N., Anzala A.O., Bwayo J.J., MacDonald K.S., Ngugi E.N., Plummer F.A. (1996). Resistance to HIV-1 infection among persistently seronegative prostitutes in Nairobi, Kenya. Lancet (London, England).

[bib26] Gottlieb G.S., Raugi D.N., Smith R.A. (2018). 90-90-90 for HIV-2? Ending the HIV-2 epidemic by enhancing care and clinical management of patients infected with HIV-2. Lancet HIV.

[bib27] Grant R.M., Lama J.R., Anderson P.L., McMahan V., Liu A.Y., Vargas L., Goicochea P., Casapía M., Guanira-Carranza J.V., Ramirez-Cardich M.E. (2010). Preexposure chemoprophylaxis for HIV prevention in men who have sex with men. N. Engl. J. Med..

[bib28] GTEx Consortium, Laboratory Data Analysis &Coordinating Center LDACC—Analysis Working Group, Statistical Methods groups—Analysis Working Group, Enhancing GTEx eGTEx groups, NIH Common Fund, He Y., Jo B., Mohammadi P., Park Y., Biospecimen Collection Source Site—NDRI (2017). Genetic effects on gene expression across human tissues. Nature.

[bib29] Gusev A., Ko A., Shi H., Bhatia G., Chung W., Penninx B.W.J.H., Jansen R., de Geus E.J.C., Boomsma D.I., Wright F.A. (2016). Integrative approaches for large-scale transcriptome-wide association studies. Nat. Genet..

[bib30] Gusev A., Mancuso N., Won H., Kousi M., Finucane H.K., Reshef Y., Song L., Safi A., Neale B.M., Schizophrenia Working Group of the Psychiatric Genomics Consortium (2018). Transcriptome-wide association study of schizophrenia and chromatin activity yields mechanistic disease insights. Nat. Genet..

[bib31] Hinrichs A.S., Karolchik D., Baertsch R., Barber G.P., Bejerano G., Clawson H., Diekhans M., Furey T.S., Harte R.A., Hsu F. (2006). The UCSC genome browser database: update 2006. Nucleic Acids Res..

[bib32] Jiang A.-P., Jiang J.-F., Guo M.-G., Jin Y.-M., Li Y.-Y., Wang J.-H. (2015). Human blood-circulating basophils capture HIV-1 and mediate viral trans-infection of CD4+ T cells. J. Virol..

[bib33] Johnson E.O., Hancock D.B., Gaddis N.C., Levy J.L., Page G., Novak S.P., Glasheen C., Saccone N.L., Rice J.P., Moreau M.P. (2015). Novel genetic locus implicated for HIV-1 acquisition with putative regulatory links to HIV replication and infectivity: a genome-wide association study. PLoS One.

[bib34] Lam M., Awasthi S., Watson H.J., Goldstein J., Panagiotaropoulou G., Trubetskoy V., Karlsson R., Frei O., Fan C.-C., De Witte W. (2020). RICOPILI: rapid imputation for COnsortias PIpeLIne. Bioinformatics.

[bib35] Lama J., Planelles V. (2007). Host factors influencing susceptibility to HIV infection and AIDS progression. Retrovirology.

[bib36] Li B., Veturi Y., Verma A., Bradford Y., Daar E.S., Gulick R.M., Riddler S.A., Robbins G.K., Lennox J.L., Haas D.W., Ritchie M.D. (2021). Tissue specificity-aware TWAS (TSA-TWAS) framework identifies novel associations with metabolic, immunologic, and virologic traits in HIV-positive adults. PLoS Genet..

[bib37] Liechti T., Kadelka C., Braun D.L., Kuster H., Böni J., Robbiani M., Günthard H.F., Trkola A. (2019). Widespread B cell perturbations in HIV-1 infection afflict naive and marginal zone B cells. J. Exp. Med..

[bib38] Limou S., Le Clerc S., Coulonges C., Carpentier W., Dina C., Delaneau O., Labib T., Taing L., Sladek R., Deveau C. (2009). Genomewide association study of an AIDS-nonprogression cohort emphasizes the role played by HLA genes (ANRS genomewide association study 02). J. Infect. Dis..

[bib39] Liu R., Paxton W.A., Choe S., Ceradini D., Martin S.R., Horuk R., MacDonald M.E., Stuhlmann H., Koup R.A., Landau N.R. (1996). Homozygous defect in HIV-1 coreceptor accounts for resistance of some multiply-exposed individuals to HIV-1 infection. Cell.

[bib40] Lopalco L. (2010). CCR5: from natural resistance to a new anti-HIV strategy. Viruses.

[bib41] Mancuso N., Freund M.K., Johnson R., Shi H., Kichaev G., Gusev A., Pasaniuc B. (2019). Probabilistic fine-mapping of transcriptome-wide association studies. Nat. Genet..

[bib42] Mancuso N., Gayther S., Gusev A., Zheng W., Penney K.L., Kote-Jarai Z., Eeles R., Freedman M., Haiman C., Pasaniuc B., PRACTICAL consortium (2018). Large-scale transcriptome-wide association study identifies new prostate cancer risk regions. Nat. Commun..

[bib43] Mancuso N., Shi H., Goddard P., Kichaev G., Gusev A., Pasaniuc B. (2017). Integrating gene expression with summary association statistics to identify genes associated with 30 complex traits. Am. J. Hum. Genet..

[bib44] Martin M.P., Carrington M. (2013). Immunogenetics of HIV disease. Immunol. Rev..

[bib45] McLaren P.J., Coulonges C., Bartha I., Lenz T.L., Deutsch A.J., Bashirova A., Buchbinder S., Carrington M.N., Cossarizza A., Dalmau J. (2015). Polymorphisms of large effect explain the majority of the host genetic contribution to variation of HIV-1 virus load. Proc. Natl. Acad. Sci. USA.

[bib46] McLaren P.J., Coulonges C., Ripke S., van den Berg L., Buchbinder S., Carrington M., Cossarizza A., Dalmau J., Deeks S.G., Delaneau O. (2013). Association study of common genetic variants and HIV-1 acquisition in 6, 300 infected cases and 7, 200 controls. PLoS Pathog..

[bib47] McLaren P.J., Fellay J. (2021). HIV-1 and human genetic variation. Nat. Rev. Genet..

[bib48] Meyer H.V. (2020). meyer-lab-cshl/plinkQC: plinkQC 0.3.2 (v0.3.2). Zenodo.

[bib49] Neale Lab (2018). http://www.nealelab.is/uk-biobank/.

[bib50] Nudel R., Wang Y., Appadurai V., Schork A.J., Buil A., Agerbo E., Bybjerg-Grauholm J., Børglum A.D., Daly M.J., Mors O. (2019). A large-scale genomic investigation of susceptibility to infection and its association with mental disorders in the Danish population. Transl. Psychiatry.

[bib51] O’Connor L.J., Price A.L. (2018). Distinguishing genetic correlation from causation across 52 diseases and complex traits. Nat. Genet..

[bib52] Patel P., Borkowf C.B., Brooks J.T., Lasry A., Lansky A., Mermin J. (2014). Estimating per-act HIV transmission risk: a systematic review. AIDS (London, England).

[bib53] Pedraza M.A., del Romero J., Roldán F., García S., Ayerbe M.C., Noriega A.R., Alcamí J. (1999). Heterosexual transmission of HIV-1 is associated with high plasma viral load levels and a positive viral isolation in the infected partner. J. Acquir. Immune Defic. Syndr..

[bib54] Jia X., Telenti A., Walker B.D., Brumme C.J., Carrington M., Carlson J.M., Graham R.R., Deeks S.G., Crawford G., International HIV Controllers Study (2010). The major genetic determinants of HIV-1 control affect HLA class I peptide presentation. Science (New York, N.Y.).

[bib55] Pers T.H., Karjalainen J.M., Chan Y., Westra H.-J., Wood A.R., Yang J., Lui J.C., Vedantam S., Gustafsson S., Esko T. (2015). Biological interpretation of genome-wide association studies using predicted gene functions. Nat. Commun..

[bib56] Powell T.R., Duarte R.R.R., Hotopf M., Hatch S.L., de Mulder Rougvie M., Breen G.D., Lewis C.M., Nixon D.F. (2020). The behavioral, cellular and immune mediators of HIV-1 acquisition: new insights from population genetics. Sci. Rep..

[bib57] Raitakari O.T., Juonala M., Rönnemaa T., Keltikangas-Järvinen L., Räsänen L., Pietikäinen M., Hutri-Kähönen N., Taittonen L., Jokinen E., Marniemi J. (2008). Cohort profile: the cardiovascular risk in Young Finns Study. Int. J. Epidemiol..

[bib58] Rodger A.J., Cambiano V., Bruun T., Vernazza P., Collins S., Degen O., Corbelli G.M., Estrada V., Geretti A.M., Beloukas A. (2019). Risk of HIV transmission through condomless sex in serodifferent gay couples with the HIV-positive partner taking suppressive antiretroviral therapy (PARTNER): final results of a multicentre, prospective, observational study. Lancet (London, England).

[bib59] Schneider T., Martinez-Martinez A., Cubillos-Rojas M., Bartrons R., Ventura F., Rosa J.L. (2018). The E3 ubiquitin ligase HERC1 controls the ERK signaling pathway targeting C-RAF for degradation. Oncotarget.

[bib60] Sultan B., Benn P., Waters L. (2014). Current perspectives in HIV post-exposure prophylaxis. HIV AIDS (Auckl).

[bib61] The Working Group on Mother-To-Child Transmission of HIV (1995). Rates of mother-to-child transmission of HIV-1 in Africa, America, and Europe: results from 13 perinatal studies. J. Acquir. Immune Defic. Syndr. Hum. Retrovirol..

[bib62] Tran B.X., Ho R.C.M., Ho C.S.H., Latkin C.A., Phan H.T., Ha G.H., Vu G.T., Ying J., Zhang M.W.B. (2019). Depression among patients with HIV/AIDS: research development and effective interventions (GAP(RESEARCH)). Int. J. Environ. Res. Public Health.

[bib63] Tropf F.C., Lee S.H., Verweij R.M., Stulp G., van der Most P.J., de Vlaming R., Bakshi A., Briley D.A., Rahal C., Hellpap R. (2017). Hidden heritability due to heterogeneity across seven populations. Nat. Hum. Behav..

[bib64] Uhlén M., Fagerberg L., Hallström B.M., Lindskog C., Oksvold P., Mardinoglu A., Sivertsson Å., Kampf C., Sjöstedt E., Asplund A. (2015). Tissue-based map of the human proteome. Science (New York, N.Y.).

[bib65] Watanabe K., Taskesen E., van Bochoven A., Posthuma D. (2017). Functional mapping and annotation of genetic associations with FUMA. Nat. Commun..

[bib66] Willer C.J., Li Y., Abecasis G.R. (2010). METAL: fast and efficient meta-analysis of genomewide association scans. Bioinformatics.

[bib67] Wright F.A., Sullivan P.F., Brooks A.I., Zou F., Sun W., Xia K., Madar V., Jansen R., Chung W., Zhou Y.H. (2014). Heritability and genomics of gene expression in peripheral blood. Nat. Genet..

[bib68] Yang X., Gabuzda D. (1999). Regulation of human immunodeficiency virus type 1 infectivity by the ERK mitogen-activated protein kinase signaling pathway. J. Virol..

[bib69] Zhang Y., Chen Y., Liu Z., Lai R. (2018). ERK is a negative feedback regulator for IFN-γ/STAT1 signaling by promoting STAT1 ubiquitination. BMC Cancer.

[bib70] Zhou T., Sun D., Li X., Ma H., Heianza Y., Qi L. (2021). Educational attainment and drinking behaviors: mendelian randomization study in UK Biobank. Mol. Psychiatry.

[bib71] Ziyatdinov A., Kim J., Prokopenko D., Privé F., Laporte F., Loh P.-R., Kraft P., Aschard H. (2021). Estimating the effective sample size in association studies of quantitative traits. G3.

